# Improving the Catalytic CO_2_ Reduction on Cs_2_AgBiBr_6_ by Halide Defect Engineering: A DFT Study

**DOI:** 10.3390/ma14102469

**Published:** 2021-05-11

**Authors:** Pengfei Chen, Yiao Huang, Zuhao Shi, Xingzhu Chen, Neng Li

**Affiliations:** 1 State Key Laboratory of Silicate Materials for Architectures, Wuhan University of Technology, Wuhan 430070, China; pengfeichen_whut@163.com (P.C.); hyahya0211@163.com (Y.H.); zuhao110317@163.com (Z.S.); chen.xz0913@gmail.com (X.C.); 2 Center of Innovation and Entrepreneurship, Wuhan University of Technology, Wuhan 430070, China; 3 Shenzhen Research Institute, Wuhan University of Technology, Shenzhen 518000, China; 4 State Center for International Cooperation on Designer Low-Carbon & Environmental Materials (CDLCEM), School of Materials Science and Engineering, Zhengzhou University, Zhengzhou 450001, China

**Keywords:** halide perovskite, CO_2_ catalytic reduction, defect engineering, computational research

## Abstract

Pb-free double halide perovskites have drawn immense attention in the potential photocatalytic application, due to the regulatable bandgap energy and nontoxicity. Herein, we first present a study for CO_2_ conversion on Pb-free halide perovskite Cs_2_AgBiBr_6_ under state-of-the-art first-principles calculation with dispersion correction. Compared with the previous CsPbBr_3_, the cell parameter of Cs_2_AgBiBr_6_ underwent only a small decrease of 3.69%. By investigating the adsorption of CO, CO_2_, NO, NO_2_, and catalytic reduction of CO_2_, we found Cs_2_AgBiBr_6_ exhibits modest adsorption ability and unsatisfied potential determining step energy of 2.68 eV in catalysis. We adopted defect engineering (Cl doping, I doping and Br-vacancy) to regulate the adsorption and CO_2_ reduction behavior. It is found that CO_2_ molecule can be chemically and preferably adsorbed on Br-vacancy doped Cs_2_AgBiBr_6_ with a negative adsorption energy of −1.16 eV. Studying the CO_2_ reduction paths on pure and defect modified Cs_2_AgBiBr_6_, Br-vacancy is proved to play a critical role in decreasing the potential determining step energy to 1.25 eV. Finally, we probe into the electronic properties and demonstrate Br-vacancy will not obviously promote the process of catalysis deactivation, as there is no formation of deep-level electronic states acting as carrier recombination center. Our findings reveal the process of gas adsorption and CO_2_ reduction on novel Pb-free Cs_2_AgBiBr_6_, and propose a potential strategy to improve the efficiency of catalytic CO_2_ conversion towards practical implementation.

## 1. Introduction

Many environmental problems such as global warming [1,2,3,4], water pollution, and natural resource depletion have spurred numerous researchers to devote concerted efforts to realizing the high-efficiency production of clean, reliable, renewable energy. Among all the proposed strategies, catalytic conversion of carbon dioxide (CO_2_), of which the released amount has far exceeded it that our ecosystem can handle, has become one of the hottest research spheres. In this regard, photo(electro)catalytic hydrogenate of CO_2_ in hydrocarbon-based “green fuels” is regarded as state-of-the-art technology. It will contribute to less reliance on fossil fuels with CO_2_ reduction production, serving as a substitute high-energy-density fuel. It will also introduce a carbon resource in the carbon-cycling which is crucial in the sustainable development of the earth [5,6]. Thus far, extensive works have witnessed great interest in semiconductors such as TiO_2_ [7,8,9,10], Cu_2_O [11,12], CdS [13,14] and g-C_3_N_4_ [15,16], with lots of experimental investigations focusing on promoting the migration rate of induced charges. However, more studies are required to uncover novel and effective ideas, properties, and aspects of the CO_2_ conversion catalysts to further advance the present state of knowledge and reaction performance to the next level.

From the view of atomic and electronic points, CO_2_ reduction can be mainly divided into two steps: forming a strong interaction between the gas molecules and the catalysts, followed by reduction reaction in the existing H^+^/e^−^ pairs. Determined by varied catalysts and reaction environment (i.e., carbonate solution, CO_2_ mixed with H_2_O vapor, pure CO_2_ gas, and so on), two to eight even numbers of the pairs will be utilized in the whole reaction leading to different final products such as CO, HCOOH, H_2_CO, CH_3_OH, and CH_4_. Nevertheless, few works give a deep insight into the relationship between CO_2_ molecular with catalysts, as the CO_2_ conversion efficiency highly depends on the surface reaction [17,18,19,20]. The successive physicochemical adsorption of the small gas molecular on to the catalyst surface guarantees effective catalysis. However, CO_2_ is not preferred to be fixed, and methods like additional energy, pressure, and temperature are often adopted to confirm the process of fixation. After that, the CO_2_ reduction encounters another challenge of first step hydrogenation: CO_2_ + e^−^ → CO^2−^ which needs a critical amount of energy to climb over a reaction barrier as large as −1.90 V vs. NHE (normal hydrogen electrode, the detail is displayed in the Electronic Supplementary Material (ESI)) in the electrochemical study. Consequently, there is a pressing need and huge quest to provide novel kinds of next-generation semiconductors with a followed optimized method to overcome the bottlenecks and cover the in-depth study of reaction mechanism to give a theoretical foundation for future researches.

Following the guidance of finding a more promising semiconductor, a great number of researches have paid attention to conventional inorganic perovskites ABO_3_ (i.e., CaTiO_3_, SrTiO_3_), which possess remarkable structural flexibility and stability with a myriad of studies [21,22,23,24,25] reporting the unique catalytic performance. Nonetheless, the large bandgap, high carriers recombination rate, small surface area, and unsatisfactory selectivity of CO_2_ reduction are still the challenges as a prominent catalyst for ABO_3_. As for the new kind of halide Pb-based perovskites ABX_3_ (i.e., CH_3_CH_2_PbI_3_, CsPbBr_3_), the toxicity of Pb^2+^ is the major bottleneck for the experimental synthesis [26,27,28,29,30,31,32].

Recently, novel Pb-free double halide perovskites A_2_BB’X_6_ have witnessed rapid advances in the past two years as a new star of catalyst. Due to the diverse collocation of atoms on the B/B’ sites, the intrinsic properties can be easily modulated. Moreover, the double perovskite structure can effectively tackle the toxicity of traditional lead halide perovskites [33]. The most typical double perovskite Cs_2_AgBiBr_6_ [34] shows a lack of toxicity compared with CsPbBr_3_, and its nanocrystal has demonstrated great potential as appealing candidates for the advanced photo(electro)catalytic applications [35,36].

**Figure 1:**
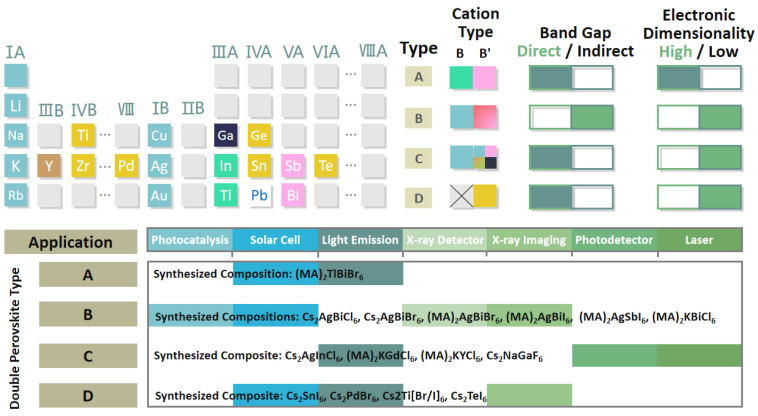
Design strategies of lead-free double perovskites by replacing Pb^2+^ with B and B’ cations, synthesis compositions, and the relationship between their electronic properties and potential applications. The concept “Electronic Dimensionality” is used to describe the ability of carriers transporting in different directions [39,40]. Reproduced with permission from [36].

Up to now, four types of double perovskites have been synthesized with different kinds of B and B’ cations (i.e., B = Li^+^, Na^+^, K^+^, Rb^+^, etc. and B’ = In^3+^, Tl^3+^, Bi^3+^, Sb^3+^, etc.), among which type A, B, and C all adopt the strategy of substituting Pb^2+^ by monovalent and trivalent ions, while type D reveals B cation vacancy and tetravalent B’ cation. Figure 1 has concluded nearly all the construction routes of double perovskites, synthesis compositions, and the electronic properties followed by potential applications. Among all the inorganic halide Pb-free perovskites, Cs_2_AgBiBr_6_ double perovskite exhibits unique semiconducting properties equipped with suitable band edges for CO_2_ reduction, high stability and nontoxicity, which can be exploited for various industrial and artificial applications in catalytic CO_2_ conversion [33,37,38]. However, there is little research reported to investigate the CO_2_ capture and conversion on the Pb-free double halide perovskites. Meanwhile, almost all the mechanism on CO_2_ conversion are established on the hypothesis that CO_2_ is the only existing gas in the reaction environmental. The question remains of whether this material be applied to the real reaction environment. Therefore, probing into the structural and electronic properties of the catalysts and using the atom-scale regulation strategy to optimize the catalytic activity is imperative. Herein, using state-of-the-art DFT calculations with dispersion corrections, Cs_2_AgBiBr_6_ is comprehensively evaluated as the potential photocatalyst for CO_2_ reduction. The capture performance of CO_2_ in the exhaust is examined and indicates the priority of the adsorption of CO_2_. In addition, the detailed CO_2_ conversion mechanism on the pure Cs_2_AgBiBr_6_ is explored, and halide defect engineering strategies (Cl, I, Br-vacancy doping) are proved to promote the process of CO_2_ reduction at different degrees. Finally, we probe into the electronic properties and demonstrate Br-vacancy will not obviously accelerate the deactivation of catalysis, as there is no formation of deep-level electronic states acting as carrier recombination center. This work reveals the process of gas adsorption and CO_2_ reduction on novel Pb-free Cs_2_AgBiBr_6_, and then propose a potential strategy to improve the efficiency of catalytic CO_2_ conversion towards practical implementation.

## 2. Computational Method

Our first-principles calculations were performed using the plane-wave pseudopotential approach under the density functional theory (DFT). And the operations were conducted within the Vienna Ab-initio Simulation Package (VASP) (5.3.5, Neng Li group, Wuhan University of Technolohy, Wuhan, China) code [41,42,43]. The generalized gradient approximation (GGA) was adopted to describe the exchange correlation functional in the form of Perdew-Burke-Ernzerhof (PBE) [44]. To more precisely describe the Van der Waals force between the substrate of the perovskite and the gas molecular, we employed the DFT-D3 empirical correction of Grimme [44]. During the optimization process, the cut-off energy was set as 250 eV for electron plane wave basis, and the convergence criteria of residual energy and force for each atom were set to 10^−4^ eV and 0.05 eV/Å. A 3 × 3 × 1 Monkhorst-Pack k-point was adopted in geometry optimization. In regard of studying the gas adsorption and CO_2_ conversion performance, a vacuum layer of 15 Å was established in the z-direction to construct the surface model. In calculating the band structure, Heyd-Scuseria-Ernzerhof (HSE) hybrid method was employed with the exact Fock exchange set to be 25%. Spin-orbit coupling (SOC) was considered, which was significant in the presence of Bi.

The catalytic reduction of CO_2_ can be divided in to proton-coupled electron transfer (PCET) steps one by one, with the possible products of CO, HCOOH, H_2_CO, CH_3_OH, and CH_4_. In each PCET step, $G_{R_{n}}$ was calculated following the Equation (1) [45]:

(1) $$G_{R_{n}}=G_{substrate+C_{1-m}O_{2-2m-l}H_{n-2l}}+mCO_{2}+lH_{2}O-G_{substrate}-G_{CO_{2}}-\frac{n}{2}G_{H_{2}}$$

where *n* represents the number of the transferred H^+^/e^−^ (the *n*^th^ PCET step), and $G_{substrate+C_{1-m}O_{2-2m-l}H_{n-2l}}$ represents the Gibbs free energy of the CO_2_ reacted with n PCET steps. The Gibbs energy can be determined as *G* = *H*^0^ − *TS* + ZPE, and the detail is displayed in ESI.

## 3. Results and Discussion

### 3.1. The Basic Crystal and Electronic Structure of Double Halide Perovskite

**Figure 2:**
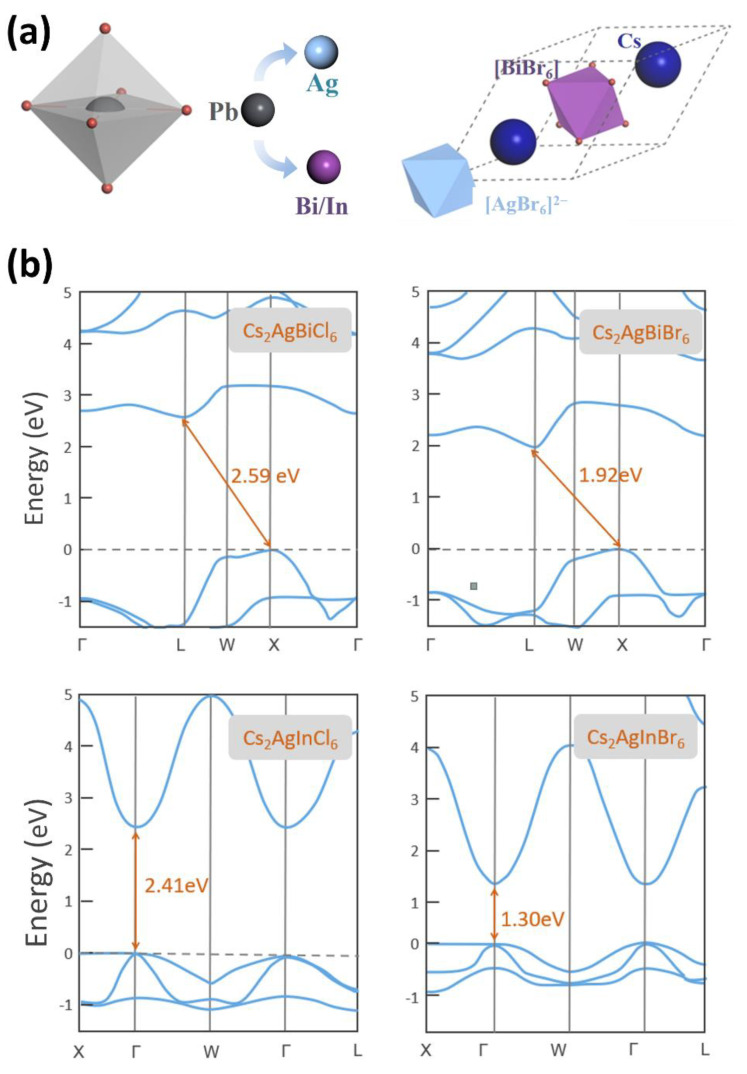
(**a**) Crystal construction strategy of Cs_2_AgBiBr_6_ based on Pb-based halide perovskite CsPbBr_3_. (**b**) The band structure of Cs_2_AgBiCl_6_, Cs_2_AgBiBr_6_, Cs_2_AglnCl_6_ and Cs_2_AglnBr_6_.

CsPbBr_3_ is one of the most typical cases of ABX_3_ halide perovskites with face-centered cubic structure, and it shows promising properties in photocatalytic(electric) reaction. However, the Pb-based perovskite faces the bottlenecks of toxicity originating from the Pb ion. Up to date, Cs_2_AgInX_6_ and Cs_2_AgBiX_6_ (X = Cl, Br) have been demonstrated to be the next generation materials for substituting Pb-based halide perovskites [46], which are suitable for utilizing visible light. Consequently, we investigated the basic crystal and the electronic band structure of Cs_2_AgInCl_6_, Cs_2_AgInBr_6_, Cs_2_AgBiCl_6_, and Cs_2_AgBiBr_6_ to find out whether these perovskites have potential in catalysis. In Figure 2a, the schematic of the substitution of Pb site and the corresponding primary cell of double halide perovskites are demonstrated. We construct the Pb-free double perovskite Cs_2_AgBiBr_6_ via the replacement of the Pb site by Ag and Bi atoms on the basis of the origin CsPbBr_3_ crystal structure. In spite of the heterovalent substitution on the Pb site, the monovalent of Ag and trivalent of Bi can maintain total charge neutrality. The radius of Ag and Bi atoms is 1.15 Å and 1.03 Å respectively, similar to the 1.19 Å of Pb atom, which can guarantee the stability of the substitutional structure. The high cubic symmetry in the primary cell of double perovskites is constructed by three different kinds of octahedrons [AgBr_6_]^2−^, [BiBr_6_]^2−^ and [CsBr_6_]^2−^. The optimized crystal structure with the lowest energy is exhibited in Figure S1 in ESI. Similar to the basic structure perovskite of CsPbBr_3_, it is a three-dimension frame with *Fm-3m* space group symmetry, formed by corner connected octahedrons, and Cs^+^ is at the octahedral interstices. For the double oxide perovskites, the rock-salt ordering is widely accepted as the ground state [47], and we believe it can also be adopted in the double halide perovskites [48]. After the lattice optimization, there is a small decrease of 3.69% in the cell parameter, compared with the original CsPbBr_3_ (11.92 Å [49]). At the same time, the bond length of Ag-Br (3.20 Å) is larger than that of Bi-Br (2.88 Å) as the Br ions undergo a light displacement toward Bi ion, contributing to the relatively stronger attractive force of Bi^3+^ than Ag^+^.

To better evaluate the feasibility as photocatalysts, the band structures of the reported double halide perovskites with the capacity in catalysis are investigated, including Cs_2_AgBiX_6_ and Cs_2_AglnX_6_ [50]. The band structures are displayed in Figure 2b. Obviously, there is an indirect bandgap in the Cs_2_AgBiX_6_ system, while the Cs_2_AglnX_6_ system possesses the direct bandgap like Pb-based CsPbCl_3_ perovskite. For the Cs_2_AgBiX_6_, the bottom of the conduction band (CBM) and the top of the valence band (VBM) are located at L and X point, respectively. The CBM and VBM in Cs_2_AgInX_6_ are both at Г point. In the above computations, the spin-orbit coupling (SOC) is considered for all the double perovskites and shrinks the bandgap of Cs_2_AgBiX_6_. When SOC is involved in Cs_2_AgBiX_6_ systems, the VBM and CBM can be more accurately determined as additional states will arise in the bandgap, leading to the downshift of the CBM [51]. As the halogen element changes from Cl to Br, the bandgap energy will undergo a decrease of 1.11 eV and 0.67 eV on Cs_2_AgBiX_6_ and Cs_2_AglnX_6_, respectively. From the point of high-efficiency solar energy ultilization, the Cs_2_AgBiCl_6_ and Cs_2_AglnCl_6_ exhibit relatively unsatisfactory ability as the utilized light wavelength is <514.52 nm and <478.76 nm, respectively. Remarkably, the band structure of Cs_2_AgInX_6_ demonstrates the impropriety as the photocatalysts. Firstly, a relatively higher recombination rate of photo-induced carriers will be induced by the direct bandgap, resulting in the decrease in redox efficiency. Secondly, due to the parity-forbidden transition at band edges in highly centrosymmetric crystal structure, the optical adsorption may be severely reduced [52]. In the Cs_2_AgBiCl_6_ system, the unique electronic structure can entirely overcome the above downsides. In addition, the comparison of typical Pb-free perovskites in respect to the lattice parameters and bandgaps is listed in Table 1. Although the organic perovskites exhibit a more satisfied bandgap for solar energy adsorption, they suffer from the weakness of instability. The type D perovskites (Figure 1) will face the challenge of decreased mobility of carriers [53,54]. Hence, the Cs_2_AgBiX_6_ is adopted as the candidate for further adsorption investigation.

### 3.2. The Carbon Dioxide Capture Capacity on Modified Cs_2_AgBiBr_6_

**Figure 3:**
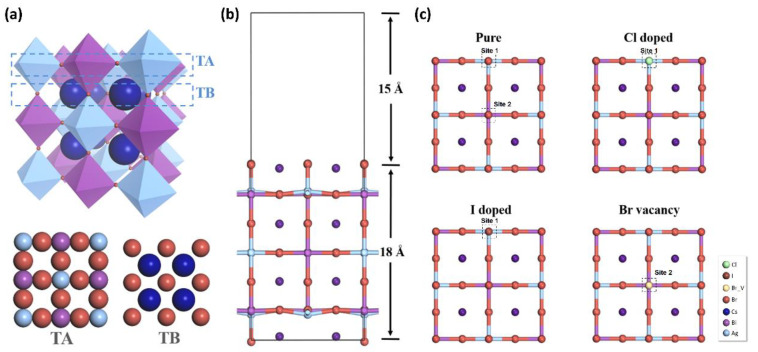
(**a**) Different terminates (TA, TB) of Cs_2_AgBiBr_6_. (**b**) The side view of the pure Cs_2_AgBiBr_6_ surface slab. (**c**) The corresponding top view of the pure surface slab (which contains two different sites of Br) as well as the Cl doped, I doped and Br-vacancy system after structure optimization.

It is worth noting that CO_2_ conversion can be applied to gas processing for factory waste gas, automobile exhaust, useless gas from a laboratory. As such, we investigated the adsorption energy of CO, CO_2_, NO, and NO_2_ to find out whether the CO_2_ adsorption is energetically preferable. In order to build a stable foundation for the Cs_2_AgBiBr_6_ framework, finding a suitable crystal termination plays an essential role for the following researches on surface catalytic reaction. Scientists have confirmed when employing room temperature in synthesis that the most stable surfaces of ABX_3_ perovskite MAPbI_3_ (tetragonal) are (001) and (110) [65,66]. While for the *Fm-3m* phase Cs_2_AgBiBr_6_, the (100) termination is equivalent to the (001) and (110) terminations of the tetragonal phase. From the view of charges, the (100) termination is nonpolar. Hence, Cs_2_AgBiBr_6_ can be treated by composed layer by layer with TA (BiBr/AgBr_3_) and TB (CsX) (Figure 3a). The (2 × 1) supercell of the optimized double halide perovskite bulk was cleaved as a (100) surface to establish the slab model, as shown in Figure 3b. The slab and vacuum layer thickness are 18 Å and 15 Å respectively. This model can simulate the surface of the perovskite [67], because the supercell will repeat continuously in the *x*-*y* plane while the vacuum layer can break the continuity in the *z*-direction. In fact, a systematic research on the termination has been given, and the TB is always favored irrespective of the CsBr availability [67]. To design a prominent photocatalytic material employed at ambient conditions, the rational selection of the terminal surfaces determines the electronic local environment on active sites. As such, we adopt TB to investigate the adsorption performance on the CO, CO_2_, NO, and NO_2_, which are the major compositions in the industry exhaust and adverse to the atmosphere. At the same time, surface modification has been revealed to promote the adsorption and catalytic performance [68,69,70,71], so we investigate the effect of Cl, I and Br-vacancy doping on Cs_2_AgBiBr_6_ surface. Displayed in Figure 3c, there are two different sites (site 1 and site 2) of Br in TB, thus defect formation energy are calculated to ascertain the energetically preferable doping sites following the Equation (2) [72]:

(2) $$E^{f}\left(D\right)=E_{tot}\left(D\right)-E_{host}\left(U\right)-\underset{i}{\sum }n_{i}\mu_{i}$$

where $E_{tot}\left(D\right)$ and $E_{host}\left(U\right)$ represents the energy of the doped system and undoped system, *n_i_* are the amounts of atoms added or removed from the host material to create the defect, $\mu_{i}$ are the chemical potentials of these atoms. Results demonstrate that Br-vacancy exhibits the smallest $E^{f}\left(D\right)$ of 3.47 eV in Site 2, which is close to the previous work in halide and oxide perovskites [73,74,75]. The Cl, I, and Br-vacancy are calculated to be relatively more stable to locate at Site 1, Site 1, and Site 2 respectively (detailed $E^{f}\left(D\right)$ information is concluded in Table S1 in ESI). The doped structures after structure optimization are displayed in Figure 3c.

**Figure 4:**
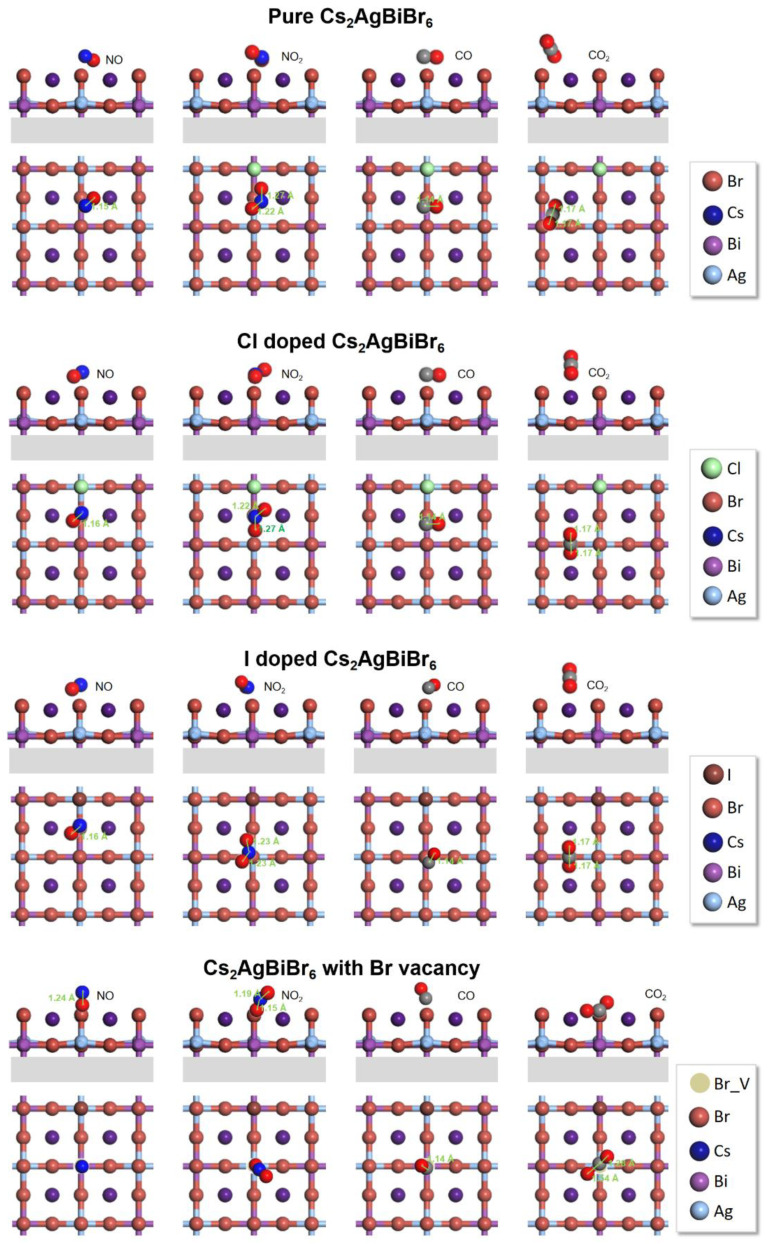
The adsorption configuration of four unique gas molecular on the pure, Cl doped, I doped, and vacancy doped Cs_2_AgBiBr_6_.

**Figure 5:**
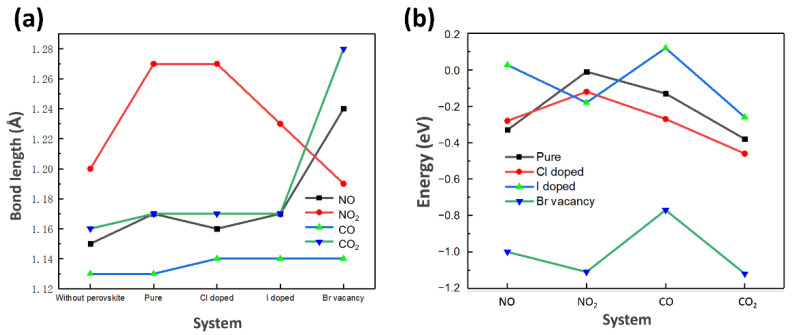
(**a**) The adsorption energy and (**b**) the bond length of four gas molecular on the pure, Cl doped, I doped and vacancy doped Cs_2_AgBiBr_6_.

The outcomes are of great importance as steady adsorption is the prerequisite of the next step in photocatalysis because the core of gas capture is changing the electronic properties of the whole system. As is demonstrated in Figure 4, the adsorption performance of CO, CO_2_, NO, NO_2_, in pure Cs_2_AgBiBr_6_ and the corresponding Cl doped, I doped, and Br-vacancy system are comprehensively investigated. To obtain the optimal structure, we consider unique adsorption sites and the orientation of gas molecular. Figure 4 displays all the optimized structure in those systems and the corresponding structural details are concluded in Table 2. We found that the O atom is attended to approach to Cs atom (as the relative high ability of O in obtaining charges and Cs in losing charges), which gives the foundation of gas molecular spontaneously adsorbed on the surface. The distance between two-O-atom molecules (NO_2_ and CO_2_) and the Cs_2_AgBiBr_6_ surface are shorter than that of one-O-atom molecules (CO and NO), and the bond length in every gas molecule is increased. Figure 5 shows the adsorption energy and bond length of each gas molecular after the structural optimization. The pure surface and the modified surface are adopted as the substrate. Nearly all of the system exhibit spontaneous adsorption of the gas molecules demonstrated from the negative value of *E_b_*_._ It is widely accepted when |*E_b_*| < 0.5 eV the adsorption process can be treated as physical adsorption, when |*E_b_*| > 0.5 eV it can be treated as chemical adsorption [76]. In this regard, the Br-vacancy perovskite can lead to the chemical adsorption of all gas molecules with the minimum value of |*E_b_*| calculated to be 0.77 eV. In Figure 5a, the pure Cs_2_AgBiBr_6_ shows the slight adsorption of NO, NO_2_, CO and CO_2_ with the range of *E_b_* from −0.38 eV to −0.01 eV. The halide dopant systems (Cl doped and I doped) both exhibit insignificant improvement in adsorption. On the other hand, the adsorption energy of NO, NO_2_, CO and CO_2_ on Br-vacancy Cs_2_AgBiBr_6_ are within −1.2 eV to −0.7 eV, much more negative than the pure and halide dopant systems. According to the previous research [77], the vacancy can accumulate massive charge on the center of the site, playing a critical role in activating adsorption species. On the other hand, the CO_2_ adsorption energy in all the Cs_2_AgBiBr_6_ systems is the lowest compared with other gas, implying the Cs_2_AgBiBr_6_ materials prefer to capture CO_2_ from the exhaust gas containing carbides and nitrides. Figure 5b demonstrates the bond length of each gas molecular on the different systems. Notably, the bonding in CO_2_ captured with the system with Br vacancy elongate most compared with the pure and dopant systems, which can illustrate the strong chemical adsorption between the CO_2_ and the vacancy site with the *E_b_* of −1.12 eV.

Moreover, the charge transfer is considered, aiming to probe into the degree of association between surface and gas molecular in respect of charges. The charge loss of pure Cs_2_AgBiBr_6_ is −0.055 eV, −0.414 eV, −0.023 eV, and −0.316 eV after the adsorption of NO, NO_2_, CO, and CO_2_, which exhibit the strong ability of NO_2_ and CO_2_ on attracting electrons.

### 3.3. The Pure Cs_2_AgBiBr_6_ for CO_2_ Catalytic Performance

**Figure 6:**
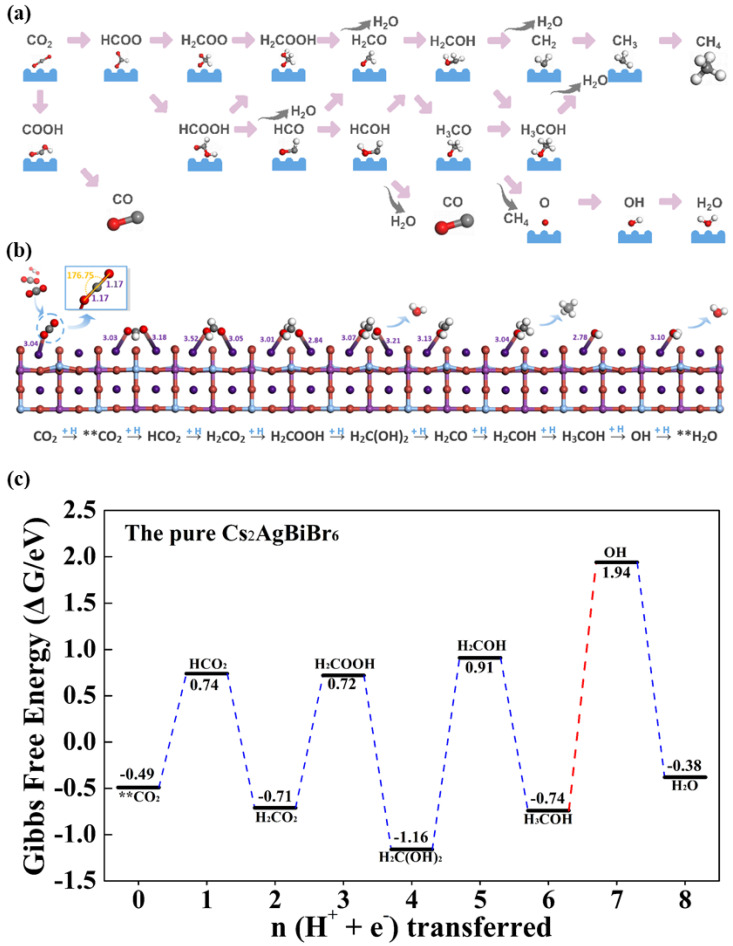
(**a**) Possible reaction path for CO_2_ reduction. (**b**) All the optimized intermediates on the pure Cs_2_AgBiBr_6_. (**c**) The calculated minimum free energy profile for the pure Cs_2_AgBiBr_6_.

Considering the end-on CO_2_ adsorption and the unique surface on Cs_2_AgBiBr_6_, the CO_2_ reduction process follows the complicated reaction pathways, as is presented in Figure 6a. For each step, the H^+^/e^−^ pairs participates in the species’ protonation either on C or O atoms. Since two adjacent Cs atoms are separated by halide atoms, the double carbon products are unable to generate. The single carbon products (i.e., CH_4_, HCOOH, CH_3_OH, CO) can be obtained via the regulation of combination sites in PCET steps and the exact amount of H^+^/e^−^ pairs participated in the reaction. If one CO_2_ molecular is only reduced by singular numbers of H^+^/e^−^ pairs, the whole system will be in an energetically unstable transition state, resulting in the next PCET step spontaneously. In this paper, we focus on the single carbon products. The CO and HCOOH molecules need two electrons in reaction, while CH_3_OH belongs to the six-electron reaction and the CH_4_ is the eight-electron product. Massive intermediates are involved in the CO_2_ reduction process. We optimized all the possible species in the pure Cs_2_AgBiBr_6_, and the most energetically favored reaction paths were obtained. The configurations of the intermediates with the lowest energy are displayed in Figure 6b, which compose the optimal reaction pathway. And the free energy profile of the whole system is displayed in Figure 6c. The reaction path is based on the largest amount of H^+^/e^−^ pairs (eight) participating in the catalysis process and enough energy applied to support overcoming the energy barrier in each PCET step, especially when an odd number of H^+^/e^−^ pairs is induced. It was found that the products of HCOOH, H_2_C(OH)_2_, H_2_COH, and CH_4_ were obtained. In the pure Cs_2_AgBiBr_6_ system, CO_2_ is firstly held by one Cs atom exposed on the TB surface, and the CO_2_ end-on model, which is fixed by two Cs atoms, has been proven to be energy-unfavorable. The first PCET step is to form highly symmetrical HCO_2_ with the free energy change of +1.23 eV. The next triple H^+^ additions mainly concentrate on one O atom beside the Cs atom, and the H_2_O is firstly released. The corresponding values of ∆*G* are −1.45 eV, +1.43 eV and −1.88 eV. Then, the remaining H_2_CO undergoes triple protonation, and the CH_4_ is released with the free energy change of +2.07 eV, −1.65 eV and +2.68 eV. Finally, OH forms H_2_O with the declined free energy change of −2.32 eV. In the pure Cs_2_AgBiBr_6_ system, the change of free energy ranges largely, implying the CO_2_ reduction process is massively exergonic, and the main product is CH_4_. The potential determining step (PDS) is H_3_COH + H^*^ → OH^*^ + CH_4_↑.This step is regarded as the crucial step for CH_4_ desorption, requiring overcoming barrier energy of 2.68 eV.

### 3.4. The Vacancy and Doping Engineering for the Improved CO_2_ Catalytic Performance

**Figure 7:**
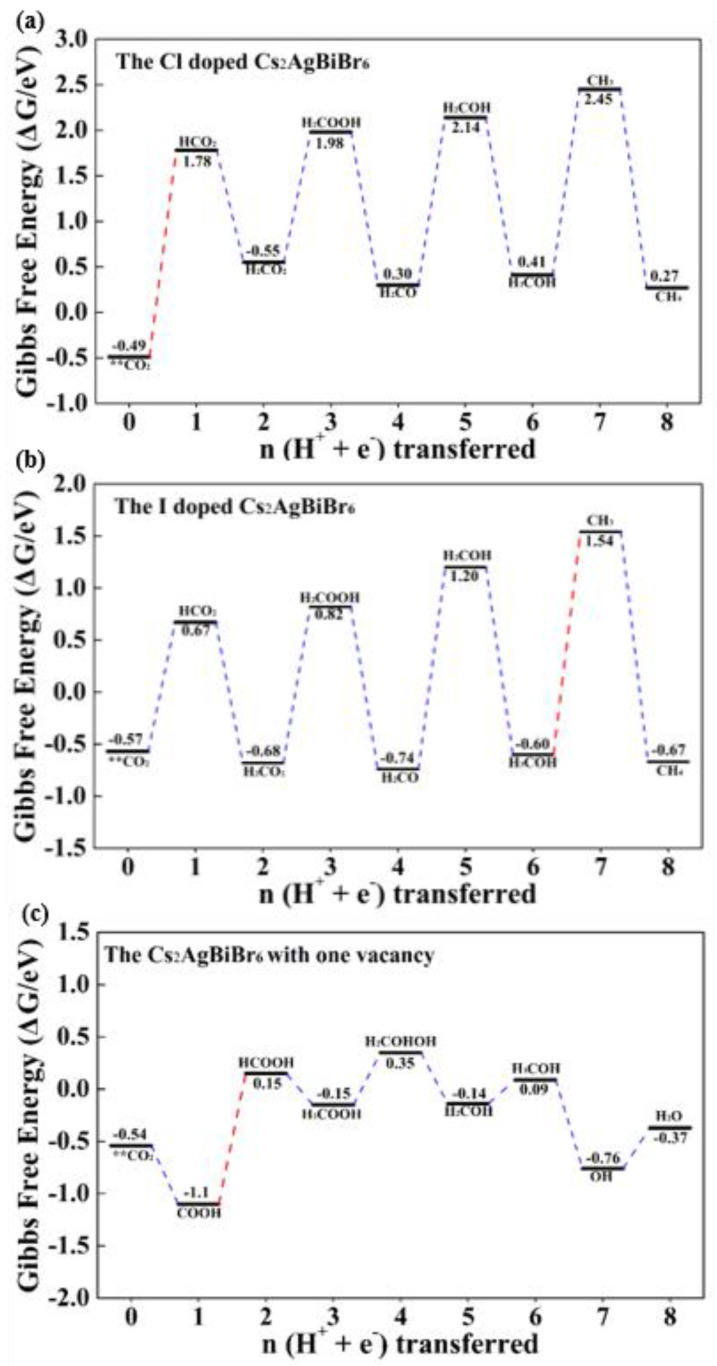
The calculated minimum free energy profile for the Cs_2_AgBiBr_6_ with (**a**) Cl dopant, (**b**) I dopant and (**c**) one vacancy on the TB.

Due to the relatively large barrier of CO_2_RR, it is imperative to modulate the intrinsic electronic properties of Cs_2_AgBiBr_6_ to enhance the catalytic activity. Vacancy and halogen doping are regarded as two electronic structure designing strategies and make a contributor to the adsorption of the intermediates. Hence, the detailed CO_2_RR process on Cs_2_AgBiBr_6_ with Cl dopant, I dopant and Br-vacancy on the TB is systematically explored (Figure 7).

In the halide-doped systems (Figure 7a,b), the optimized CO_2_ reduction pathway follows **CO_2_ → HCO_2_ → H_2_CO_2_ → H_2_COOH → H_2_CO → H_2_COH → H_3_COH → CH_3_ → CH_4_, demonstrating the similarity to the pure Cs_2_AgBiBr_6_. However, when the H_3_COH species is protonated, the H^+^/e^−^ pairs are inclined to add on O atom. In the Cl and I dopant systems, the H_2_O is firstly released with the increasing free energy of 2.04 eV and 2.14 eV. The value of the seventh PCET step is smaller than that of +2.68 eV in the pure system, which can better illustrate that the halide dopant plays a significant role in the decrease of the active barrier. The final product, CH_4_, is generated from the attack of H^+^/e^−^ pairs to the CH_3_ species. Owing to the high activation in CH_3_ species, this step results in the high stability of the reactant CH_4_ with the distinct downhill free energy change of −2.13 eV and −2.21 eV. CH_4_ is still captured by the Cs site, and the adsorption energy of CH_4_ on Cl and I dopant systems are −0.14 eV and −1.02 eV, respectively. For the I dopant system, the adsorption energy is too low, which will indicate the suppressed process of desorption. In comparison, the moderate adsorption energy of CH_4_ on the Cl dopant system demonstrates the advantages in adsorption and desorption for CO_2_RR. The PDSs in Cl and I dopant systems are respectively **CO_2_ → HCO_2_ and H_3_COH → CH_3_, requiring the energy input of 2.27 eV and 2.14 eV. In spite of the fact that halide doping further activates the species in CO_2_RR process, the improved efficiency is insignificant, and the value of PDS is still above 2 eV.

Considering the vacancies often formed in experiments, continued computation via the optimized free energy profile for catalytic CO_2_ reduction on the Cs_2_AgBiBr_6_ with one vacancy was carried out and the reaction path is shown in Figure 7c. Distinguished from the pure and halide dopant system, the pathway on Br-vacancy Cs_2_AgBiBr_6_ follows **CO_2_ → COOH → HCOOH → H_2_COOH → H_2_COHOH → H_2_COH → H_3_COH → OH+CH_4_ → H_2_O. The first two PCET steps produce the new intermediates HCOOH with the maximal barrier of 1.25 eV. In the next two steps, H^+^/e^−^ pairs are prior to occupy the C atom and then add to the O atom. After the formation of H_2_O, there are still two H atoms connected with C atom, thus the next two PCET steps prompt the release of CH_4_. Obviously, the free energy range of the whole intermediates in CO_2_ reduction maintains in a small scale. The PDS is the process of COOH to form HCOOH (1.25 eV), and this value is comparable to the Au catalyst [78] and sulfur-doped g-C_3_N_4_ [79] for CO_2_ reduction. This relative low barrier of PDS predicts the smooth CO_2_ conversion in Cs_2_AgBiBr_6_ with vacancies.

**Figure 8:**
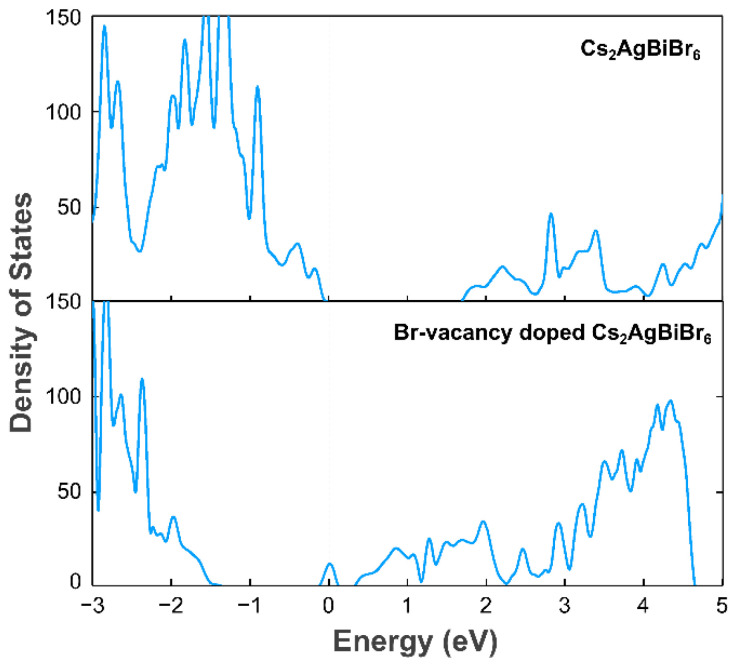
DOS plot for Cs_2_AgBiBr_6_ and Br-vacancy doped Cs_2_AgBiBr_6._

To reveal the effect of Br-vacancy on the electronic property of Cs_2_AgBiBr_6_, we calculated the density of states (DOS) for pure and Br-vacancy Cs_2_AgBiBr_6_ surface using the method of GGA-PBE. From Figure 8, it can be deduced that the forming of Br-vacancy can move the Fermi level from VBM to near the CBM, which is consistent with previous work on studying the O-vacancy and Cl-vacancy [80,81,82]. As the peak introduced by defective states is sharp and separated from the relatively delocalized electrons in the conduction band, the exceeding electrons brought by vacancy are localized. It widely acknowledged that deep-level impurity can act as the recombination center for carriers, which will lead to the deactivation of catalysts. The vacancy adopted in this work introduces the extra defective electronic states located very close to the CBM, which can be defined as shallow doping energy level, thus not expected to accelerate the catalysis deactivation. In addition, although the vacancy decreases the bandgap energy, such a slight decrease in bandgap will have minor effects on the conversion of carriers [83].

## 4. Conclusions

In summary, employing the well-resolved DFT calculations, we concentrate on the comprehensive investigation in the Cs_2_AgBiBr_6_ as the novel CO_2_ reduction catalyst. Based on the structure of CsPbBr_3_, Ag^+^/Bi^3+^ and Ag^+^/In^3+^ are adopted to substitute Pb^2+^ to realize the objection on Pb-free, keeping the crystal stable and the charge balance. The Cs_2_AgBiBr_6_, of which the bandgap is calculated to be 1.92 eV, is determined to be the most potential material for CO_2_RR. Further studies on CO, CO_2_, NO, NO_2_, gas capture proved Cs_2_AgBiBr_6_ a suitable material for CO_2_ adsorption and the doping and vacancy-doped systems still demonstrate the simultaneous tendency for CO_2_ preference. Moreover, the detailed CO_2_RR pathway on the pure, Cl-doped, I-doped and Br-vacancy Cs_2_AgBiBr_6_ are studied with the judgment of Gibbs free energy. The vacancy-doping system could significantly promote the procedure with the potential determining step (PDS) of 1.25 eV, compared with 2.68 eV of pure system, 2.27 eV of Cl-doped system and 2.14 eV of I-doped system. Further investigation of the Cs_2_AgBiBr_6_ with Br-vacancy reveals that the vacancy will not obviously promote the process of catalysis deactivation, as there is no formation of deep-level electronic states acting as carrier recombination center. In this regard, this work paves a potential avenue in demystifying the defect modification mechanism on lead-free halide double perovskites, which will lay a foundation for defect engineering in CO_2_RR photocatalysts toward a host of environmental and energetic applications.

## Figures and Tables

**Table 1:** Structure information and bandgaps of typical Pb-free halide perovskites obtained from theoretical first-principle and experimental studies.

Perovskites	Space Group	Lattice Parameters (Å)	Band Gap (eV)		Ref.
			Theory	Experiment	
Cs_2_AgBiCl_6_	Fm3-m	10.51 (This work)	2.59 (This work)	2.41 (Ref. [55])	
Cs_2_AgBiBr_6_	Fm3-m	11.48 (This work)	1.92 (This work)	2.02 (Ref. [56])	
Cs_2_AgInCl_6_	Fm3-m	10.53 (This work)	2.41 (This work)	2.1 (Ref. [57])	
Cs_2_AgInBr_6_	Fm3-m	10.12 (This work)	1.30 (This work)	1.17 (Ref. [58])	
Cs_2_SnI_6_	Fm3-m	11.6276	1.3	1.26	[59]
		11.6276	1.6	1.62	[54]
		11.65	-	-	[60]
Cs_2_TiBr_6_ Cs_2_TiBr_6_(@C_60_)	Fm3-m	10.92	0.89 1.01	- -	[61]
Cs_2_TiI_6_	Fm3-m	11.67	0.79	-	[32]
CsRbSnI_6_	Pmn2_1_	a = 8.2608 b = 12.1507 c = 8.7913	1.58	-	[62]
(CH_3_NH_3_)_2_AgBiBr_6_	Fm3m	11.6370	2.02	2.02	[63]
(CH_3_NH_3_)_2_KBiCl_6_	R3m	a = 7.8372 c =20.9938	3.02	3.04	[64]

**Table 2:** Conclusion of structure information before and after gas molecular adsorbed on the substrate.

Pervskite	Gas	*E_b_* (eV)	Bond Length of Gas Molecular (Å)		Bond Angle of Gas Molecular (º)	
			Original	Adsorbed	Original	Adsorbed
Pure	NO	−0.33	1.15	1.17	/	/
	NO_2_	−0.01	1.20	1.27, 1.22	134.3	127.47
	CO	−0.13	1.13	1.13	/	/
	CO_2_	−0.38	1.16	1.17, 1.17	180.0	176.75
Cl doped	NO	−0.28	1.15	1.16	/	/
	NO_2_	−0.12	1.20	1.27, 1.22	134.3	127.47
	CO	−0.27	1.13	1.14	/	/
	CO_2_	−0.46	1.16	1.17, 1.17	180.0	179.55
I doped	NO	0.027	1.15	1.17	/	/
	NO_2_	−0.18	1.20	1.23, 1.23	134.3	126.63
	CO	0.12	1.13	1.14	/	/
	CO_2_	−0.26	1.16	1.17, 1.17	180.0	
Br vacancy	NO	−1.00	1.15	1.24	/	/
	NO_2_	−1.11	1.20	1.19, 1.15	134.3	149.71
	CO	−0.77	1.13	1.14	/	/
	CO_2_	−1.12	1.16	1.28, 1.24	180.0	143.15

## Data Availability

The data presented in this study are available on request from the corresponding author.

## Supplementary Materials

The following are available online at https://www.mdpi.com/article/10.3390/ma14102469/s1, The illustration of NHE (Normal Hydrogen Electrode), The calculation of Gibbs free energy, Figure S1: Scheme of the optimized crystal structure and two types of Br, which can be doped by Cl and I, Table S1: $E^{f}\left(D\right)$ values calculated for Cl, I, Br-vacancy doped in Site 1 and Site 2.
