# Supplementary material for: Improving the Catalytic CO2 Reduction on Cs2AgBiBr6 by Halide Defect Engineering: A DFT Study
Source: Materials (Basel). 2021 May 11;14(10):2469. doi: 10.3390/ma14102469 (PMC8151533; doi:10.3390/ma14102469)
Supplement: Supplementary file 1 [file materials-14-02469-s001.zip › materials-1197744-supplementary.pdf]

Supporting Information

Improving the Catalytic CO_2 reduction on $Cs_2AgBiBr_6$ by Halide Defect Engineering: A DFT Study

Pengfei Chen,† Yiao Huang,† Zuhao Shi, Xingzhu Chen, Neng Li*

P. Chen, Y. Huang, Z. Shi, X. Chen, Prof. N. Li
State Key Laboratory of Silicate Materials for Architectures, Wuhan University of Technology, Hubei, 430070, China
E-mail: lineng@whut.edu.cn

P. Chen, Y. Huang, Z. Shi
Center of Innovation and Entrepreneurship, Wuhan University of Technology, Hubei, 430070, China
Homepage: https://www.x-mol.com/groups/WUT_CIE.com

Z. Shi, X. Chen, Prof. N. Li
Shenzhen Research Institute of Wuhan University of Technology, Shenzhen 518000, China

Prof. N. Li
State Center for International Cooperation on Designer Low-Carbon & Environmental Materials (CDLCEM), School of Materials Science and Engineering, Zhengzhou University, Zhengzhou 450001, Henan, China

† These authors contributed equally to this work.

The illustration of NHE (Normal Hydrogen Electrode)

The Standard Hydrogen Electrode is often abbreviated as SHE or may be known as normal hydrogen electrode (NHE), and its standard electrode potential is declared to be 0 at a temperature of 298K. This is because it acts as a reference for comparison with any other electrode. Nowadays NHE is widely adopted in a wide range of researches [1-4] which represent SHE actually.

The redox half cell of SHE is where the following reaction takes place:

$2H^+ (aq) + 2e^- \rightarrow H_2 (g)$

The reaction given above generally takes place on a platinum electrode. The pressure of the hydrogen gas present in this half cell equals 1 bar.**The calculation of Gibbs free energy**

The fundamental thermodynamic relation states that:

$$G^0 = H^0 - TS \quad (1)$$

at temperatures greater than 0 K and constant pressure, enthalpy for a given temperature (here T = 298.15 K) can be expressed in terms of *H0* and the heat capacity, C_P:

$$H = H^0 + \int C_p dT \quad (2)$$

in addition, the entropy term can be expressed as the sum of the translational, rotational,
vibrational, and electronic contributions as to:

$$S = S_t + S_r + S_v + S_e \quad (3)$$

and also, intrinsic zero point energy (ZPE) and extrinsic dispersion (*D*) corrections can be included to finally obtain:

$$G = H^0 + \int C_p\, dT - T(S_t + S_r + S_v + S_e) + ZPE + D \quad (4)$$

When computing the Gibbs free energy variation between two states named as 1 and 2, and applying eqn. (4), ΔG results in:

$$\Delta G_{21} = H_2^0 + \int C_{p,2}\, dT - T\big(S_{t,2} + S_{r,2} + S_{v,2} + S_{e,2}\big) + ZPE_2 + D_2 - H_1^0 - \int C_{p,1}\, dT - T\big(S_{t,1} + S_{r,1} + S_{v,1} + S_{e,1}\big) + ZPE_1 - D_1 \quad (5)$$

or simply:

$$\Delta G_{21} = H_{21}^0 + \Delta \int (C_p)_{21} dT - T\Delta S_{21} + \Delta ZPE_{21} + \Delta D_{21} \quad (6)$$

Over this last equation, some approximations can be applied:

1. At the fundamental electronic level: $S_e \approx 0$
2. For gases, translational, rotational, and vibrational entropy terms have contributions that might not be neglected, and therefore: $S = S_t + S_r + S_v$
3. For solids and adsorbates, both $S_t \approx 0$ and $S_r \approx 0$ and therefore: $S = S_v$
4. Since $\int C_p\, dT$ is almost negligible and $\Delta \int C_p\, dT \approx 0$, no thermal corrections for the enthalpy have been taken into account for the ΔG calculation.

Based on the fundamental thermodynamic relation states analysis, the computing of the ΔG change for the CH_4 (g) chemisorption on a clean MXene surface (binding energy), can be expressed as:

$$\Delta G(\mathrm{CH_4 \cdots Cs_2AgBiBr_6}) = G(\mathrm{CH_4 \cdots Cs_2AgBiBr_6}) - G(\mathrm{Cs_2AgBiBr_6}) - G(\mathrm{CH_4})$$

(7)

where:

$$G(\mathrm{CH_4 \cdots Cs_2AgBiBr_6}) = H^0(\mathrm{CH_4 \cdots Cs_2AgBiBr_6}) - \mathrm{T}S_v(\mathrm{CH_4 \cdots Cs_2AgBiBr_6}) + \\ + \mathrm{ZPE}(\mathrm{CH_4 \cdots Cs_2AgBiBr_6}) + D(\mathrm{CH_4 \cdots Cs_2AgBiBr_6})$$

(7.1)

$$G(\mathrm{Cs_2AgBiBr_6}) = H^0(\mathrm{Cs_2AgBiBr_6}) - \mathrm{T}S_v(\mathrm{Cs_2AgBiBr_6}) + \mathrm{ZPE}(\mathrm{Cs_2AgBiBr_6}) + D(\mathrm{Cs_2AgBiBr_6}) \quad (7.2)$$

$$G(\mathrm{CH_4}) = H^0(\mathrm{CH_4}) - \mathrm{T}S_t(\mathrm{CH_4}) - \mathrm{T}S_r(\mathrm{CH_4}) - \mathrm{T}S_v(\mathrm{CH_4}) + \mathrm{ZPE}(\mathrm{CH_4}) + \\ + D(\mathrm{CH_4})$$

(7.3)

Finally, this can be also extended to the calculation of the Gibbs free reaction energy, and therefore eqn. (8) has been applied to calculate ΔG_R, where n is the number of H^+/e^- pairs transferred and m the number of CH_4 molecules released, if applicable. In such a context, the chemical potential of the H^+/e^- pair has the half value of the chemical potential of the dihydrogen (H_2) molecule [see eqn. (9)],[1] when working at standard hydrogen electrode (SHE) conditions,

i.e.$\mathrm{pH} = 0\ \ \mathrm{f(H_2)} = 101{,}325\ \mathrm{Pa}$, and $U = 0$ V, being f(H_2) and U the fugacity of H_2 and the external potential applied, respectively:

$$\Delta G_R = \mathrm{G(CO_2 \cdots Cs_2AgBiBr_6) - n\,G(CH_4) - G(Cs_2AgBiBr_6) - G(CO_2) - n/2G(H_2)} \quad (8)$$

For n = 0, $\Delta G_R = \Delta G_b$, binding Gibbs free energy in ($CO_2 \cdots Cs_2AgBiBr_6$) (8.1)

$$\mu(H^+/e^-) = {}^1\!/_2\,\mu(H_2) \quad (9)$$

Pseudo-potentials applied

Nitrogen: PAW_PBE N 08Apr2002

Hydrogen: PAW_PBE H 15Jun2001

Oxygen: PAW_PBE O 08Apr2002

Carbon: PAW_PBE C 08Apr2002

Bismuth: PAW_PBE Bi 08Apr2002

Silver: PAW_PBE Ag 02Apr2005

Caesium: PAW_PBE Cs_sv 08Apr2002

Chlorine: PAW_PBE Cl 06Sep2000

Bromine: PAW_PBE Br 06Sep2000

Iodine: PAW_PBE I 08Apr2002

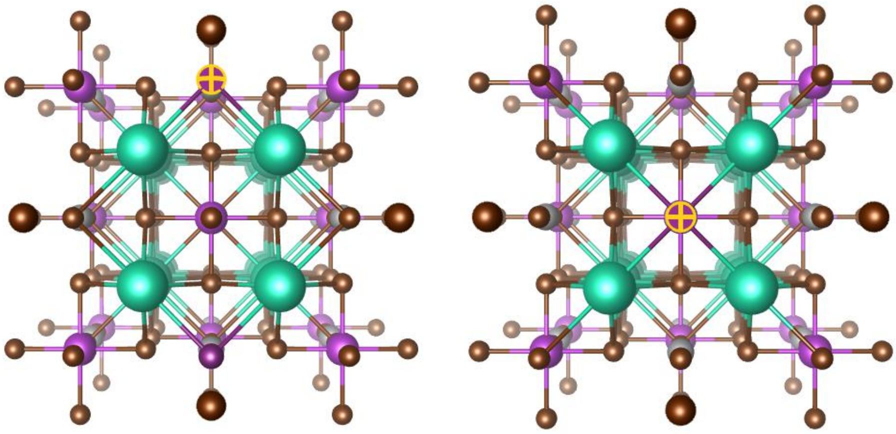

Figure S1 Scheme of the optimized crystal structure and two types of Br, which can be doped by Cl and I. (Brown: Br, green: Cs, grey: Ag, violet: Bi)

Table S1 $E^f(D)$ values calculated for Cl, I, Br-vacancy doped in Site 1 and Site 2.

Sites	Cl doping	I doping	Br-vacancy
Site 1	0.93 eV	1.06 eV	3.61 eV
Site 2	0.96 eV	1.10 eV	3.47 eV

References

1. Ponce de León, C., In situ anodic generation of hydrogen peroxide. *Nat. Catal.* **2020,** 3, (2), 96-97.
2. Huang, B.; Hart, J. N., DFT study of various tungstates for photocatalytic water splitting. *Phys. Chem. Chem. Phys.* **2020,** 22, (3), 1727-1737.
3. Azofra, L. M.; Sun, C.; Cavallo, L.; MacFarlane, D. R., Feasibility of N_2 Binding and Reduction to Ammonia on Fe-Deposited MoS_2 2D Sheets: A DFT Study. *Chem.-.Eur. J.* **2017,** 23, (34), 8275-8279.

4. Skúlason, E.; Karlberg, G. S.; Rossmeisl, J.; Bligaard, T.; Greeley, J.; Jónsson, H.; Nørskov, J. K., Density functional theory calculations for the hydrogen evolution reaction in an electrochemical double layer on the Pt(111) electrode. *Phys. Chem. Chem. Phys.* **2007,** 9, (25), 3241-3250.
